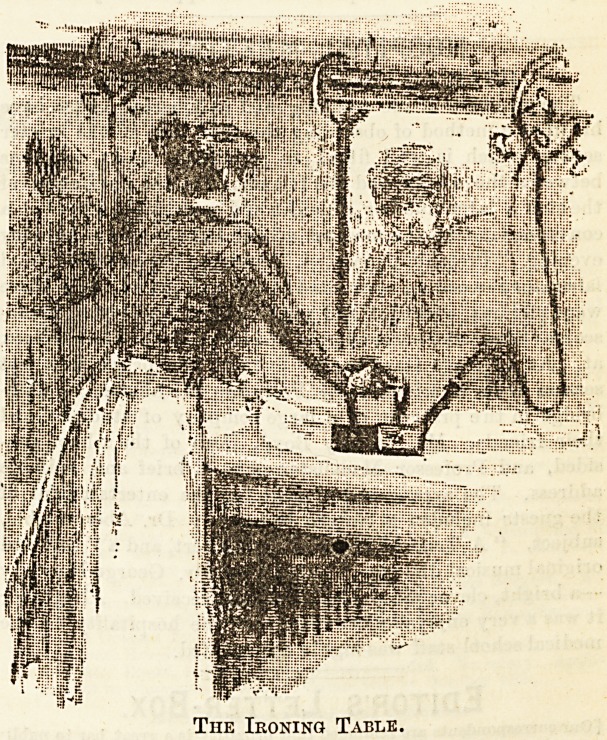# Practical Departments

**Published:** 1894-10-06

**Authors:** 


### PRACTICAL DEPARTMENTS.

### HOSPITAL LAUNDRIES (*continued*).

Steam mangles have now been in use in nearly all large laundries, institutional or otherwise, for a number of years. The "box-mangle" is the one most frequently seen. A different variety is in favour in the Troy laundries—the "Duplex" mangle—and it is a machine which undeniably

Shirt Ironer.

turns out an enormous quantity of most excellent work. It is intended to dry and iron the goods at the same time, and is provided with two heated cylinders and iron rollers covered with woollen blanketing and muslin, the latter serving both to protect the hems of tablecloths, sheets, &c.,

The Ironing Table.

and to absorb moisture. "Feeding aprons" carry the materials to be manipulated to the cylinders, while a "receiving apron" on the opposite side takes them from the heated cylinders and deposits them on the folding table. It is really necessary to see one of these machines in operation to fully appreciate the amount of work they got through, and also the "finish" which is imparted to the materials.

The "Shirt-ironer" which we here illustrate, by permission of Mr. Armstrong, the representative in London of the Troy Laundry Machinery Company, is a useful little machine, and doubtless effects considerable saving of labour. It is easy to work.

The introduction of gas as a means of heating the hand irons has been of inestimable comfort to laundry workers. Our second illustration shows a corner of the long ironing table in the Troy laundry at Ealing. Gas is laid on within easy range, and the jet itself is inside the iron. A thoroughly even heat is thus kept up, and there is none of the inevitable loss of time which must take place where irons have to be perpetually reheated. Where collars and cuffs are to be hand-ironed a clever arrangement of pressure is brought into use, resulting in the turning out of beautifully-finished and glossy starched linen.

We should not omit to mention that Mr. Armstrong especially recommends the practice of "boiling" the starch, contrary, we believe, to the usual custom, at least in English laundries. Mr. Armstrong contends that a very much purer substance is thus obtained, with better results all round. Strong "starch kettles" are to be had for this purpose.

In speaking strongly of the importance of fitting a hospital laundry with the best machinery obtainable, we do not overlook the fact that the initial outlay must be a very considerable one, and that hospital funds are more often than not in an unsatisfactory state; but, if we may judge from the length of time such machinery as that at the London Hospital has been in use, it is an item of expenditure which well repays what has been spent upon it. The saving of time, labour, and consequent wages, too, increases in proportion to the amount of machinery used, while the work is better done, and the task of supervision and administration is lessened. Hospital authorities will certainly do well to adopt modern improvements in this respect whenever opportunity offers.

## Figures and Tables

**Figure f1:**
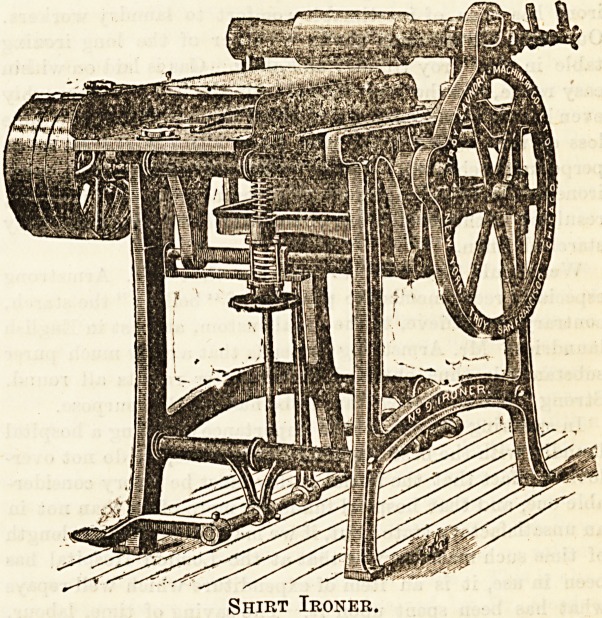


**Figure f2:**